# Allyl isothiocyanate induced stress response in *Caenorhabditis elegans*

**DOI:** 10.1186/1756-0500-4-502

**Published:** 2011-11-17

**Authors:** AkalRachna K Saini, Robert T Tyler, Youn Young Shim, Martin JT Reaney

**Affiliations:** 1 51 Campus Drive, Department of Food and Bioproduct Sciences, University of Saskatchewan, Saskatoon, Saskatchewan S7N 5A8, Canada; 2 343-111 Research Drive, Helix BioPharma Corp, Saskatoon, Saskatchewan S7N 3R2, Canada; 3 51 Campus Drive, Department of Plant Sciences, University of Saskatchewan, Saskatoon, Saskatchewan S7N 5A8, Canada

**Keywords:** *Brassica*, myrosinase, glucosinolate, HSP70, toxicity, ELISA

## Abstract

**Background:**

Allyl isothiocyanate (AITC) from mustard is cytotoxic; however the mechanism of its toxicity is unknown. We examined the effects of AITC on heat shock protein (HSP) 70 expression in *Caenorhabditis elegans*. We also examined factors affecting the production of AITC from its precursor, sinigrin, a glucosinolate, in ground *Brassica juncea *cv. Vulcan seed as mustard has some potential as a biopesticide.

**Findings:**

An assay to determine the concentration of AITC in ground mustard seed was improved to allow the measurement of AITC release in the first minutes after exposure of ground mustard seed to water. Using this assay, we determined that temperatures above 67°C decreased sinigrin conversion to AITC in hydrated ground *B. juncea *seed. A pH near 6.0 was found to be necessary for AITC release. RT-qPCR revealed no significant change in HSP70A mRNA expression at low concentrations of AITC (< 0.1 μM). However, treatment with higher concentrations (> 1.0 μM) resulted in a four- to five-fold increase in expression. A HSP70 ELISA showed that AITC toxicity in *C. elegans *was ameliorated by the presence of ground seed from low sinigrin *B. junce*a cv. Arrid.

**Conclusions:**

• AITC induced toxicity in *C. elegans*, as measured by HSP70 expression.

• Conditions required for the conversion of sinigrin to AITC in ground *B. juncea *seed were determined.

• The use of *C. elegans *as a bioassay to test AITC or mustard biopesticide efficacy is discussed.

## Background

Plant seeds have evolved a broad spectrum of natural defense mechanisms, such as physical and chemical barriers. Mustard species mitigate a wide range of biotic challenges using the glucosinolate-myrosinase system, also referred to as 'The Mustard Bomb' [1]. Glucosinolates (glucoraphanin, glucoerucin, gluconasturtiin, sinigrin, glucotropaeolin, glucoraphenin, glucoraphasatin, glucomoringin and glucobrassicin) are hydrolysed by the enzyme myrosinase (thioglucosidase) to produce an aglycone, which undergoes spontaneous non-enzymatic rearrangement to produce organic isothiocyanates, thiocyanates, nitriles, epithionitriles, oxazolidinethiones and organic cyanates [2-4].

Many glucosinolate products, including allyl isothiocyanate (AITC), are of interest because of their broad spectra of biological activities. For example, the toxicity of Indian mustard and AITC were demonstrated on masked chafer Beetle larvae [5]. The biopesticidal [6,7], fungicidal [6,8], antibiotic [9,10] and nematocidal [11,12] properties of AITC also have been studied.

*Caenorhabditis elegans *has been used as a model system to study stress responses. The stress response in *C. elegans *and most other organisms is characterized by the rapid expression of heat shock proteins (HSPs). There is extensive evidence in the literature that HSPs play an important role in the tolerance of an organism to a variety of biotic and abiotic stresses that are not immediately lethal, by maintaining cell function and survival during stress or by facilitating recovery after removal of a stressor [13,14].

During cellular stress, members of the highly conserved and ubiquitous 70 kDa heat shock protein (HSP70) family are involved in preventing protein aggregation and refolding of denatured proteins [14]. HSP70 is involved in regulating the heat shock response and other stresses through mitogen-activated protein kinase (MAPK) signaling [15]. Heschl and Baillie [16] characterized the HSP70 multigene family in *C. elegans*.

Interest in using *Brassica *material as a biopesticide requires a robust assay to determine AITC production and a bioassay to determine sample effectiveness. In the present study, we developed a method for measuring AITC in ground mustard shortly after the addition of water. In addition, we report various factors affecting AITC release *in vitro*. The effect of AITC and ground mustard on *C. elegans *was determined by measuring the transcription and translation of nematode HSP70 as an indicator of stress.

## Materials and methods

*Brassica juncea *cv. Arrid was obtained from Derek Potts of Viterra, Saskatoon, SK. *B. juncea *cv. Vulcan and *Sinapis alba *seed were obtained from Kevin Falk, Agriculture and Agri-Food Canada, Saskatoon Research Centre, Saskatoon, SK. Seed was produced on plots near Saskatoon in 2006.

### Modifying the AITC ground seed assay

The method to extract AITC from ground seed and determine its concentration is essentially that of Raquet [17]. Glucosinolates in ground seed are converted to isothiocyanates by continuously stirring 5 g of seed in 100 mL of water at 37°C for 2 h. AITC in ground seed is then recovered by adding 20 mL of 95% ethanol and a few boiling chips. Sixty millilitres of the distillate was collected in a flask containing 10 mL of 33.5% ammonium hydroxide solution and 20 mL of 0.1 N silver nitrate was added. The final volume was adjusted to 100 mL with distilled water and incubated overnight in the dark at room temperature. The resulting black precipitate was removed by filtration with Whatman grade No. 4 filter paper (GE Health Care, Piscataway, NJ) and two titrations were performed, each using 50 mL of this filtrate. The filtrate (50 mL) was acidified with 5 mL of concentrated nitric acid (analytical grade, Sigma-Aldrich, Oakville, ON, Canada) and was titrated with 0.1N ammonium thiocyanate (analytical grade, Sigma-Aldrich) after adding 5 mL of 8% FeNH_4_(SO_4_)_2_.12H_2_O indicator (Sigma-Aldrich). Percent volatile oil was calculated by:

$$\text{Volatile}\;\text{oil}\;\left(\%\right)=\frac{\left[\left(\text{mL}\times \text{N}\right)\text{AgN}{\text{O}}_{3}-2\left(\text{mL}\times \text{N}\right)\;\text{N}{\text{H}}_{4}\text{SCN}\right]\times 0.04958\times 100}{\text{Weight}\;\text{of}\;\text{sample}\;\left(\text{g}\right)}$$

We examined the effects of varying the incubation time, solvent and temperature on AITC release by ground *B. juncea *seed. The effects of various durations (0-5 min at 30 s intervals and 5, 15, 30, 60 and 120 min), temperatures (7 to 97°C) and solvents (water, 0.02 N sodium hydroxide, 0.02 N hydrochloric acid, 0.02 N acetic acid) on percent volatile oil released were examined. Four replicates of each treatment were performed.

### *C. elegans *culture and AITC treatment

*C. elegans *N2 strain was grown on 10% bacteriological agar (Sigma-Aldrich) layered with 1 mL of autoclaved 1% (w/v) Baker's yeast in sterile 10-mm diameter plates. Cultures were incubated in the dark at room temperature and sub-cultured to fresh plates every 15 days.

Two-week-old cultures of *C. elegans *were treated with 0-10 μM of commercially prepared AITC (Sigma-Aldrich) and/or 0.0-144.5 μg/mL *B. juncea *cv. Arrid ground seed and incubated in the dark at room temperature for 2 h. After treatment and incubation, cultures were centrifuged at 400 × g for 10 min at 4°C and the pellet was stored at -80°C pending further analysis.

### RNA isolation and quantitative RT-PCR analysis

Total RNA was extracted from *C. elegans *lysates stored at -80°C using an RNeasy Mini kit (Qiagen Inc., Mississauga, ON, Canada) according to the manufacturer's instructions. The integrity of RNA was confirmed by agarose gel electrophoresis and RNA was quantified using a Nano drop spectrophotometer (Thermo, Fisher Scientific, Ottawa, ON, Canada). Following DNase treatment, the mRNA was reverse transcribed at 42°C for 30 min using the QuantiTect Reverse Transcription kit (Qiagen Inc.) as per the manufacturer's instructions. This cDNA was used for quantitative real-time RT-PCR (qRT-PCR) analysis for the expression of HSP70A (GenBank Accession No. M18540) using the QuantiFast SYBR Green kit (Qiagen Inc.) as per the manufacturer's instructions. The glyceraldehyde-3-phosphate dehydrogenase gene (GAPDH; GenBank Accession X04818) was used as the reference housekeeping gene. The reactions were performed using the following primer pairs: 5'-ATGAGTAAGCATAACGCTGTT-3' and 5'-ACAGTGTTATGTGGGTTCATG-3' for a 200 bp HSP70A fragment and 5'-AACCATGAGAAGTACGAC-3' and 5'-CTGTCTTCTGGGTTGCGG-3' for a 212 bp GAPDH fragment. A negative control reaction consisted of all the components of the reaction mixture except cDNA. Real-time PCR analysis was performed using a MX3005P LightCycler (Stratagene, La Jolla, CA, USA) and the following program: initial denaturation at 94°C for 5 min; 45 cycles of denaturation at 94°C for 15 s; annealing at 57°C for 30 s; and elongation at 68°C for 60 sec. Relative expression levels were calculated after correction for expression of GAPDH using MxPro software.

### Quantification of HSP70 by an enzyme-linked immunosorbant assay (ELISA)

As HSP70A is a cytoplasmic protein known to be expressed in response to stress or toxicity, studies on the effects of AITC on *C. elegans *focused on expression of this protein. A 52 amino acid residue fragment at the C-terminal end of HSP70A (GenBank accession AAA28078) showed > 98% identity to the human HSP70 (GenBank accession NP_005337). A goat antibody raised against the human peptide of this peptide fragment was purchased from AbCam (Cambridge, MA, USA).

*C. elegans *samples stored at -80°C were ground to a fine powder under liquid nitrogen using a sterile mortar and pestle. Ground tissues were incubated in 200 μL of freshly prepared lysis buffer (50 mM Tris-HCl, pH 7.4; 150 mM NaCl; 1% NP-40; 1 mM phenylmethylsulfonyl fluoride; 5 μg/mL antipain; 5 μg/mL aprotinin; 5 μg/mL leupeptin; 7.5% polyvinylpolypyrrolidone) on ice for 30 min. The lysate was stored at -80°C until further use. Total protein was quantified using the Bradford dye-binding assay (Bio-Rad Laboratories, Hercules, CA, USA) as per the manufacturer's instructions.

Levels of induced HSP70 were measured in protein extracts from AITC-treated worms using a sandwich ELISA kit (Stressgen Biotechnologies, Ann Arbor, MI, USA) as per the manufacturer's instructions. Absorbance at 450 nm was measured using a NOVOstar microplate reader (BMG Labtech, Durham, NC, USA). The HSP70 concentrations of the samples were quantified by interpolating absorbance readings from the standard curve.

## Results

### Effects of temperature and pH on AITC release

**Figure 1:**
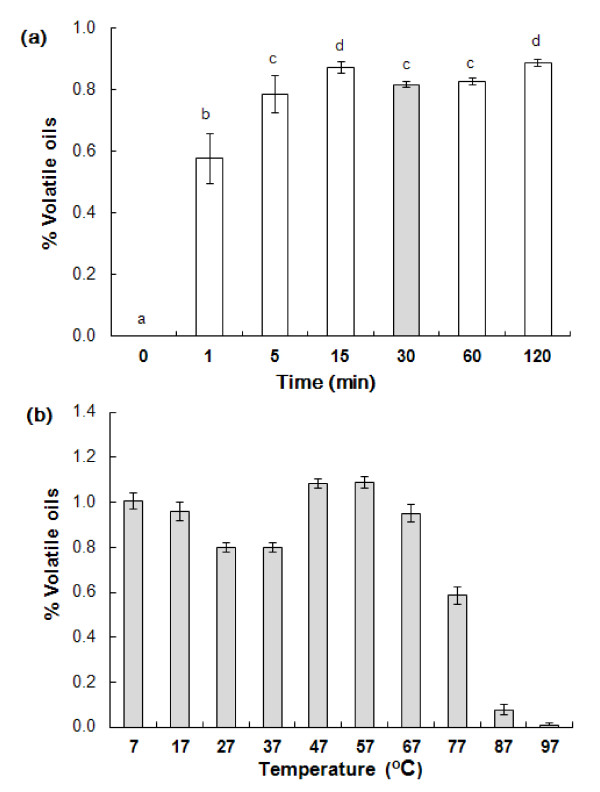
**(a) Near maximum AITC production was attained in 5 g *B. juncea *cv Vulcan ground seed after one min at 27°C**. Four replicates were performed. Data were analyzed by one-way ANOVA, significant differences are indicated with different letters (p < 0.05). (b) Effect of temperature and incubation time on the yield of AITC from 5 g ground *B. juncea *seed.

Modifications were made to the method of Raquet [17], which is used by industry to measure total AITC in ground mustard products. Initially, we examined if myrosinase could be inactivated rapidly so that incubation could be reduced from the two hours specified. High levels of AITC production in ground *B. juncea *cv. Vulcan seeds suspended in water at 27°C were observed after one minute (Figure 1a).

We next determined the effects of temperature and pH on AITC production in ground *B. juncea *cv. Vulcan seed. The rate of AITC release decreased at temperatures greater than 67°C (Figure 1b). Some AITC was released from the ground seed at 77°C, but two hours at this temperature did not result in AITC production equivalent to that achieved at lower temperatures. AITC release was almost completely inhibited at temperatures above 87°C.

AITC concentration was determined after reacting ground seed with 0.02 N solutions of NaOH, CH_3_COOH and HCl at pH 13.0, 3.0 and 1.5, respectively, and the results were compared with those for the control (water at pH 6.0). The reactions were conducted at 27°C for 5 min. An increase or decrease in pH resulted in a reduction in enzyme activity as compared to the control (Table 1), indicating that the enzyme had a near-neutral optimum. Some AITC release occurred from ground seed incubated in the presence of NaOH or CH_3_COOH. Returning the pH to 6.0 in these two reactions resulted in the release of additional AITC (Table 1). Hydrochloric acid irreversibly blocked further AITC release, however. Glucosinolates were not adversely affected by acetic or hydrochloric acid or sodium hydroxide as the addition of 1 g of ground *S. alba *seed (a source of enzyme, but devoid of substrate, i.e. sinigrin) to the neutralized treatments released AITC, presumably from unreacted sinigrin.

**Table 1:** Effect of pH on the yield of AITC.

Alkali/Acid	pH	% volatile oil	Neutralized to pH 6.0 (% volatile oil)	Addition of 1 g powdered *S. alba *seed (% volatile oil)
Water (Control)	6.0 ± 0.4	0.793 ± 0.061	ND^1^	0.821 ± 0.078
0.02 N NaOH	13.0 ± 1.0	0.079 ± 0.005	0.158 ± 0.014	0.872 ± 0.081
0.02 N CH_3_COOH	3.0 ± 0.2	0.039 ± 0.003	0.178 ± 0.021	1.150 ± 0.161
0.02 N HCl	1.5 ± 0.1	0.118 ± 0.021	0.079 ± 0.006	1.189 ± 0.125

^1^Not detected

### Exposure to AITC induces expression of HSP70A in *C. elegans*

Nematodes were visibly injured by treatment with AITC, as was indicated by their movement. Nematodes were actively moving at all concentrations of AITC below 10 μM, but higher concentrations appeared to be lethal as their movement stopped.

**Figure 2:**
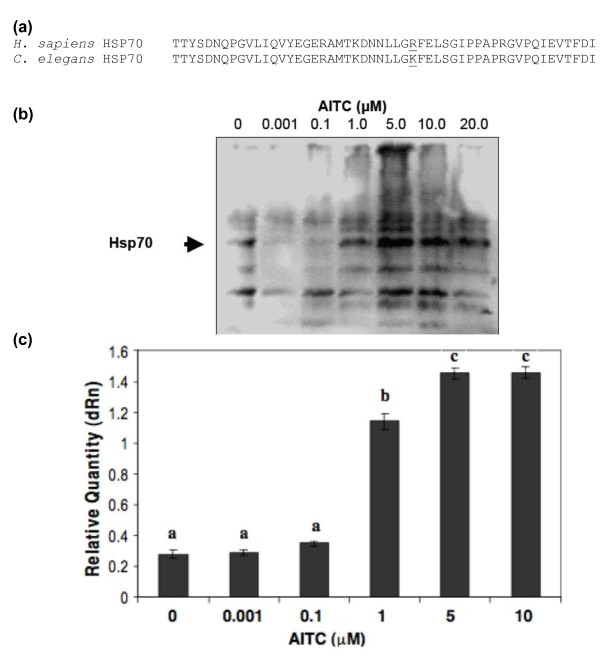
**AITC induces expression of HSP70 in *C. elegans *when treated with 0-10 μM AITC for 2 h at room temperature**. (a) Human HSP70 (Accession No. NM_005346), residues 429-480, show 98% homology with *C. elegans *HSP70A (Accession No. M18540). A monoclonal antibody directed against a synthetic peptide for this region of the human protein was obtained to detect the *C. elegans *homologue (b) Increasing concentrations of AITC induce greater expression of HSP70, indicated with arrowhead. Proteins in cell lysates from AITC treated *C. elegans *cultures were separated by SDS-PAGE and HSP70 protein detected using the anti-HSP70 monoclonal antibody. (c) Increasing concentrations of AITC induce HSP70A mRNA expression in *C. elegans*, as determined by qRT-PCR. Bars on the columns represent standard deviations of three values. Data was analyzed by one-way ANOVA. Values marked with different letters were significantly (p < 0.01).

No significant changes, compared to the control, in the levels of HSP70A transcripts were detected by qRT-PCR at concentrations of AITC of 0.1 μM or lower. However, *C. elegans *treated with higher concentrations of AITC (1 to 10 μM) responded with a 4- to 5-fold increase in expression of HSP70A transcripts (Figure 2). These results further confirm that AITC induces stress in *C. elegans*.

HSP70 protein in *C. elegans *exposed to different concentrations (0-20 μM) of AITC for two hours at room temperature was observed by Western blotting followed by immunodetection with anti-HSP70 monoclonal antibody raised against human peptide (Figure 2). *C. elegans *treated with AITC at concentrations greater than 1 μM showed an increase in HSP70 expression.

### Ground mustard seed toxicity is due to AITC

The above experiments suggest that concentrations of AITC greater than 1 μM caused toxicity in *C. elegans*. However, it is important to know whether other compounds in ground mustard seed, other than AITC, could also contribute to toxicity. We used an ELISA to determine the effect of ground mustard seed on AITC-induced HSP70 expression.

*B. juncea *cv. Arrid contains greatly reduced levels of sinigrin, the precursor of AITC, and was used to treat *C. elegans*, with or without additional AITC. The dose was based on AITC levels of 1% of the total ground seed. Total protein extract from *C. elegans *treated with AITC (0-10 μM), *B. juncea *cv. Arrid (0-144.5 μg) or AITC (0-10 μM) + *B. juncea *cv. Arrid (0-144.5 μg) for 2 h was analyzed by ELISA.

As expected, AITC alone resulted in significant increases in the expression of HSP70 protein (Table 2). ELISA was able to detect HSP70 expression at 0.001 μM. Only the highest concentration of ground *B. juncea *cv. Arrid seed (144.5 μg) induced the expression of HSP70 proteins. *C. elegans *treated with both AITC and ground seed showed increased expression of HSP70 proteins, but the levels of induction were lower than those observed with an equivalent concentration of AITC alone.

**Table 2:** Effect *of B.juncea *cv. Arrid on AITC toxicity

AITC (μM)	Ground Seed (μg)	HSP70 (ng/mg)^1^
0.0	0.0	0.0 ± 0.0^a^
0.001	-	74.0 ± 4.1^b^
-	0.01445	0.0 ± 0.0^a^
0.001	0.01445	2.1 ± 4.0^a^
0.1	-	75.2 ± 5.4^b^
-	1.445	0.0 ± 0.0^a^
0.1	1.445	8.1 ± 4.2^c^
1.0	-	98.1 ± 4.8^d^
-	14.45	0.0 ± 0.0^a^
1.0	14.45	17.0 ± 4.1^e^
5.0	-	80.0 ± 6.1^d^
-	72.25	0.0 ± 0.0^a^
5.0	72.25	54.5 ± 5.1^f^
10	-	79.1 ± 5.5^d^
-	144.5	30.2 ± 4.2^g^
10.0	144.5	76.2 ± 5.1^d^

^1^*C. elegans *cultures were exposed to AITC (0-10 μM), *B. juncea *cv. Arrid (0-144.5 μg) or AITC (0-10 μM) + *B. juncea *cv. Arrid (0-144.5 μg) for 2 h at room temperature and the total protein extract was analyzed by ELISA for the presence of HSP70 protein.

Data was analyzed by two-way ANOVA (n = 4).

Values marked with different letters were significantly different from each other (p < 0.01).

## Discussion

Condiment mustard has a characteristic flavour, which is contributed by glucosinolate hydrolysis products, AITC in particular. Use of mustard, as a biopesticide is an application based on the toxicity of AITC. Other applications where a broad spectrum of toxicity and volatility are advantageous, e.g. in antimicrobial preparations, could also take advantage of AITC. A convenient assay for AITC production in ground seed and sensitive bioassays of its toxicity are required for the development of these products. The present work could help the mustard industry develop the burgeoning biopesticide market.

The current industrial AITC assay method requires a two-hour incubation at 37°C prior to steam distillation and titration. In our hands, five minutes incubation time at room temperature and near-neutral pH were sufficient to hydrolyze much of the glucosinolate to AITC. Therefore, samples with normal myrosinase activity will quickly release AITC from sinigrin. However, even a greatly degraded myrosinase activity could release all of the AITC from endogenous sinigrin in ground seed in a two-hour period. The spice trade assay is designed to determine the total release of AITC from mustard but samples with diminished enzyme activity may not be detected by this method. Shorter duration of incubation is necessary to determine variations in the rate of AITC release among samples.

Attempts also were made to inhibit or reduce AITC release by manipulating the pH of the extraction medium. The results indicated that myrosinase required a pH of 6.0 for optimum activity, and that a change in pH did not impact the substrate but altered the release of AITC. While it is known that acidic conditions may hydrolyze glucosinolates to produce nitriles and other products [18], the conditions employed in many of these conversions included a high temperature (100°C). Therefore, our observation that the substrate concentration was not impacted during a brief treatment of the meal with acid followed by neutralization is not unexpected. Eylen et al. [19] studied the thermal and pressure stability of myrosinase at temperatures of 60-75°C and pressures from ambient to 1000 MPa. Myrosinase was found to be stable at 600 MPa pressure at temperatures up to 60°C. At low pressures, there was an antagonistic effect between temperature and pressure. They suggested that the high-pressure stability of myrosinase may present a valuable alternative to thermal treatment if one wants to retain myrosinase activity. The conditions for inactivation of myrosinase using microwave energy also were studied with a response surface design. Microwave irradiation at 2450 MHz for 2.5 min inhibited myrosinase and decomposed glucosinolates [20].

In the present investigation, *C. elegans *was used to study AITC-mediated stress or toxicity by investigating expression of HSP70. RT-qPCR revealed dose-dependent expression of HSP70 in *C. elegans *due to exposure to AITC. As there may be more than a single mode of action for glucosinolate toxicity, and toxicity may be due to other compounds present in ground mustard seed, a sensitive ELISA method was developed to detect HSP70 in *C. elegans *treated with AITC and/or ground seed. Together, these results indicate that 1) ground mustard seed toxicity is likely due to AITC, and 2) ground mustard seed acts as an AITC antagonist or absorbent, reducing its toxicity in *C. elegans*. These findings suggest that HSP70 could be used as a universal tool for studying mustard toxicity.

If AITC and mustard are to be used more widely in new pesticide products, questions will arise regarding both the risks to non-target organisms and the efficacy of the compound on target organisms. The regulatory environment will often require extensive testing for compounds in the environment, yet the presence of a compound does not imply either impact or toxicity. The need for expensive analytical tests may slow the introduction of new products with potent toxicity. Conversely, simple and robust tests may aid in the development of suitable policy and regulations regarding the deployment of the compound or material. The HSP approach of using a sentinel organism could be a useful tool to allow the determination of toxicity *in situ *and to potentially enable the deployment of mustard as a biopesticide.

## Abbreviations

AITC: allyl isothiocyanate; qRT-PCR: quantitative reverse transcription polymerase chain reaction; ELISA: enzyme-linked immunosorbant assay

## Competing interests

Dr. Reaney has been awarded patent number WO 2009/079792. Portions of this work were included in Ms. Saini's MSc thesis.

## Authors' contributions

Ms. S designed and performed the experiments presented in this manuscript. Ms. S also wrote the initial draft of the manuscript. All three authors were involved with the analysis and interpretation of the data.
